# c-Rel Controls Multiple Discrete Steps in the Thymic Development of Foxp3^+^ CD4 Regulatory T Cells

**DOI:** 10.1371/journal.pone.0026851

**Published:** 2011-10-31

**Authors:** George Grigoriadis, Ajithkumar Vasanthakumar, Ashish Banerjee, Raelene Grumont, Sarah Overall, Paul Gleeson, Frances Shannon, Steve Gerondakis

**Affiliations:** 1 Centre for Immunology, Burnet Institute, Melbourne, Australia; 2 Australian Centre for Blood Diseases and Department of Clinical Hematology, Monash University, Alfred Medical Research and Education Precinct, Melbourne, Australia; 3 Bio21, University of Melbourne, Parkville, Australia; 4 The John Curtin School of Medical Research, Australian National University, Canberra City, Australia; 5 Department of Immunology, Monash University, Alfred Medical Research and Education Precinct, Melbourne, Australia; University of Crete, Greece

## Abstract

The development of natural Foxp3^+^ CD4 regulatory T cells (nTregs) proceeds via two steps that involve the initial antigen dependent generation of CD25^+^GITR^hi^Foxp3^−^CD4^+^ nTreg precursors followed by the cytokine induction of Foxp3. Using mutant mouse models that lack c-Rel, the critical NF-κB transcription factor required for nTreg differentiation, we establish that c-Rel regulates both of these developmental steps. c-Rel controls the generation of nTreg precursors via a haplo-insufficient mechanism, indicating that this step is highly sensitive to c-Rel levels. However, maintenance of c-Rel in an inactive state in nTreg precursors demonstrates that it is not required for a constitutive function in these cells. While the subsequent IL-2 induction of Foxp3 in nTreg precursors requires c-Rel, this developmental transition does not coincide with the nuclear expression of c-Rel. Collectively, our results support a model of nTreg differentiation in which c-Rel generates a permissive state for *foxp3* transcription during the development of nTreg precursors that influences the subsequent IL-2 dependent induction of Foxp3 without a need for c-Rel reactivation.

## Introduction

Regulatory T cells (Tregs), CD25^+^CD4^+^ T lymphocytes that express the Foxp3 transcription factor, restrict the extent and duration of T cell mediated immune responses [1], as well as maintain peripheral self-tolerance by suppressing auto-reactive T cells that escape negative selection in the thymus [2], [3], [4]. The importance of Tregs in the inhibition of self-reactive T cells is best illustrated by the severe autoimmune disease that afflict humans and mice with developmental or functional defects in this T cell lineage [5]. The majority of Foxp3^+^CD4 T cells develop in the thymus soon after birth and are referred to as natural Treg or nTreg cells [6]. Peripheral Foxp3^−^CD25^−^CD4 T cells can also be converted by TGFβ into Foxp3^+^CD25^+^CD4^+^ T cells [7], [8], with these TGFβ inducible Tregs (iTregs) possessing immune suppressive properties akin to those of nTregs [9].

The development of nTregs occurs via a two-step process reliant on multiple intracellular pathways activated by a combination of T cell receptor (TCR), CD28 and cytokine receptor mediated signals [10], [11]. The primary developmental step involves antigen selected CD4^+^CD8^+^ thymocytes differentiating into CD25^+^GITR^hi^Foxp3^−^CD4^+^ cells, a population highly enriched for nTreg precursors [11]. This initial step in nTreg development is dependent on signals generated by TCR bound self-peptide/MHC class II complexes [12] and B7 ligand/CD28 interactions [13]. nTregs emerge from a pool of antigen selected thymocytes that express TCRs with a relatively high affinity for self antigens [14]. This developmental requirement for nTregs differs from the fate of conventional CD4 T cells expressing higher affinity TCRs, which are eliminated by negative selection [15]. The subsequent conversion of nTreg precursors into functional nTregs involves the IL-2 and/or IL-15 induction of Foxp3 expression [11], a step dependent on the regulation of *foxp3* transcription by a number of different transcription factors.

Foxp3 serves an essential role maintaining a pattern of gene expression responsible for the immune suppressive properties of Tregs [16], whereas the differentiation of nTregs is dictated by other transcription factors [1]. c-Rel, an NF-κB family member, is a transcription factor that was recently shown to control nTreg development [17], [18], [19]. While mice lacking c-Rel have ∼15% of normal thymic nTreg numbers [17], the remaining *c-rel^−/−^* Foxp3^+^nTregs possess relatively normal immune suppressive properties [17]. c-Rel is thought to control nTreg development in several ways [18], [19], [20]. A recent study that reported the frequency of CD25^+^GITR^hi^Foxp3^−^CD4^+^ thymocytes is reduced in *c-rel^−/−^* mice [21], indicates that c-Rel is required for the generation of nTreg precursors. Defects in TCR [22] and CD28 [21] signaling also reduce nTreg precursor numbers [21] , a phenotype shared with *c-rel^−/−^* mice that is consistent with c-Rel being activated in CD4 T cells through both of these receptors [23]. It remains unclear whether c-Rel is induced by TCR and/or CD28 signals during this step in nTreg development. c-Rel has also been implicated in the control of Foxp3 expression, with the mechanism by which it might regulate *foxp3* transcription a topic of considerable debate. In one model, c-Rel has been proposed to promote *foxp3* transcription by binding to a CD28 response element in CNS3, a conserved region within intron 1 of the *foxp3* gene that is required for the generation of normal thymic nTreg numbers [20]. c-Rel also binds to sites within a region of the *foxp3* 5′ un-translated region that encompasses a conserved CpG island [18], the demethylation of which is necessary for the heritable maintenance of *foxp3* transcription in nTregs [18]. This finding has led to suggestions that c-Rel might regulate *foxp3* transcription by promoting the demethylation of the CpG island. Finally, c-Rel was shown to activate *foxp3* promoter reporter constructs by binding to unique Rel-NFAT sites that appear to be required for the formation of an nTreg specific “enhanceosome” [19]. Our finding that Foxp3 expression is normal in the remaining *c-rel^−/−^* thymic nTregs [17] indicates that c-Rel is probably dispensable for constitutive Foxp3 expression. Instead, the collective results linking c-Rel to Foxp3 expression are compatible with a model in which c-Rel initiates a transcriptional program that leads to stable Foxp3 expression in nTregs [24], [25]. This putative role has led to c-Rel being dubbed a ‘pioneer’ transcription factor [24].

To better understand how c-Rel promotes nTreg development, we generated *c-rel^−/−^* mice that express a functional GFP-Foxp3 fusion protein encoded by a *foxp3* gene that is subject to normal transcriptional control [26]. These *c-rel^−/−^foxp3^gfp^* mice permitted a careful analysis of the role(s) c-Rel serves during the two key steps in nTreg development, including a functional assessment of whether c-Rel controls Foxp3 expression. Our study confirms that c-Rel is important for generating normal numbers of nTreg precursors and reveals that this developmental step is regulated in a *c-rel* copy dependent fashion. We also demonstrate that c-Rel is required for efficient IL-2 and IL-15 dependent conversion of Foxp3^−^nTreg precursors into Foxp3^+^nTregs. While c-Rel is highly expressed in CD25^+^GITR^hi^Foxp3^−^CD4^+^ cells, its localisation to the cytoplasm of these cells is indicative of c-Rel being in an inactive state and suggests that c-Rel is necessary for the generation but not the maintenance of nTreg precursors. The inability of *c-rel^−/−^* nTreg precursors to efficiently up-regulate Foxp3 coincides with a signalling defect downstream of IL-2R that results in impaired STAT5 phosphorylation following IL-2 stimulation of *c-rel^−/−^* nTreg precursors. Despite IL-2 plus IL-7 co-stimulation restoring STAT5 phosphorylation in *c-rel^−/−^* nTreg precursors, a failure of this cytokine combination to rescue the Foxp3 expression defect in these cells indicates that c-Rel must control the function of multiple components in the IL-2 signal transduction pathway required for the optimal induction of Foxp3. Finally, the finding that IL-2 induction of Foxp3 in nTreg precursors occurs in the absence of detectable nuclear c-Rel expression leads us to propose that c-Rel establishes a permissive state for *foxp3* transcription prior to the formation of nTreg precursors that influences the subsequent cytokine dependent induction of Foxp3, without a need to re-activate c-Rel.

## Results

### c-Rel promotes the development of thymic nTreg precursors via a haplo-insufficient mechanism that is independent of cell survival

**Figure 1:**
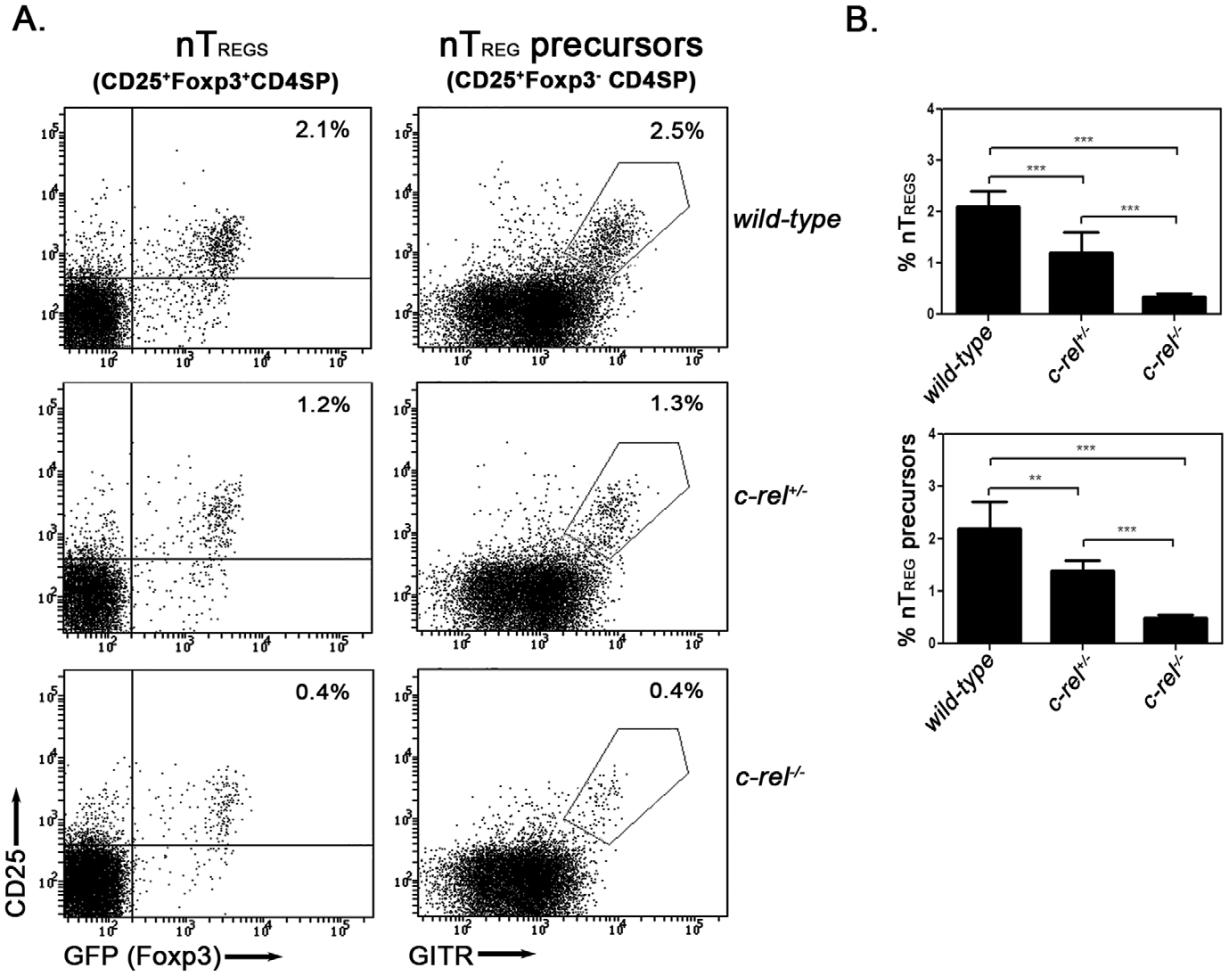
nTreg precursor populations in c-Rel deficient mice. Single cell suspensions from the thymi of *foxp3^gfp^*, *c-rel^+/−^foxp3^gfp^* and *c-rel^−/−^foxp3^gfp^* mice stained with antibodies for CD4, CD8, CD25 and GITR were examined by flow cytometry. Representative dot plots from one of six independent experiments are shown with the percentage of cells in the relevant quadrants indicated. (A) Profiles of CD25 versus Foxp3 expression (gated on CD4 SP cells) and CD25 versus GITR expression (gated on Foxp3^−^CD4SP cells) for thymocyte populations in *wt*, *c-rel^+/−^* and *c-rel^−/−^* mice. (B) Percentages of thymic nTregs (CD25^+^Foxp3^+^CD4SP) and nTreg precursors (CD25^+^Foxp3^−^CD4SP) in *wt*, *c-rel^+/−^* and *c-rel^−/−^* mice. The data represents the mean values (±SEM with ANOVA p value <0.0001) compiled from six independent experiments described in panel A.

Although c-Rel is expressed at high levels in Foxp3^+^ nTregs, its cytoplasmic sequestration in these cells, a hallmark of transcriptional inactivity indicates that c-Rel is dispensable for the maintenance of Foxp3^+^ cells and instead is necessary for nTreg differentiation [17]. While the generation of nTregs comprises a two-step process that initially involves TCR and CD28 signalling, followed by an IL-2 dependent step, it remained unclear exactly when c-Rel is required during this developmental process. We addressed this question by utilizing the *foxp3^gfp^* reporter mouse [26] to examine c-Rel expression and its sub-cellular localization during nTreg development, plus the consequences an absence of c-Rel has on the different steps of nTreg differentiation. Initially nTreg development was compared in *foxp3^gfp^* and *c-rel^−/−^foxp3^gfp^* mice. Consistent with the properties displayed by the parental *c-rel^−/−^* [17] and *foxp3^gfp^* mice [26], the only phenotypic defect observed in the different mature thymocyte populations of the *c-rel^−/−^foxp3^gfp^* mutant was a reduction in Foxp3^+^CD4 regulatory T cells (Fig. 1A and 1B), which were reduced from 2.1% to 0.4% of CD4 SP thymocytes. A comparison of thymic CD25^+^GITR^hi^Foxp3^−^(GFP^−^)CD4^+^ cells in 4 to 6-week old *foxp3^gfp^* and *c-rel^−/−^foxp3^gfp^* mice, a population highly enriched in *wild-type* (*wt*) mice for nTreg precursors [11], revealed that the percentage (2.5% and 0.4% respectively in *wt* and *c-rel^−/−^* mice; Fig. 1A and 1B) and absolute number (Table S1) of these cells was markedly reduced in *c-rel^−/−^foxp3^gfp^* mice. This finding, which agrees with a recent report [21], establishes that both the Foxp3^−^ nTreg precursor and Foxp3^+^nTreg populations are much smaller in the absence of c-Rel.

The thymic CD25^+^GITR^hi^Foxp3^−^CD4^+^ and CD25^+^GITR^hi^Foxp3^+^CD4^+^ populations in *c-rel^+/−^foxp3^gfp^* mice were found to be of an intermediate size when compared with age and sex matched *foxp3^gfp^* and *c-rel^−/−^foxp3^gfp^* animals (Fig. 1A and 1B). Consistent with published studies showing c-Rel is not essential for the development of other thymocytes [27], [28], [29], the size of populations that include conventional CD4 and CD8 SP thymocytes, NK and NKT cells was normal in *c-rel^+/−^* and *c-rel^−/−^* mice (results not shown). This finding that the copy number of *c-rel* influences the sizes of the nTreg precursor and nTreg populations, establishes that c-Rel regulates nTreg development in a haplo-insufficient manner.

**Figure 2:**
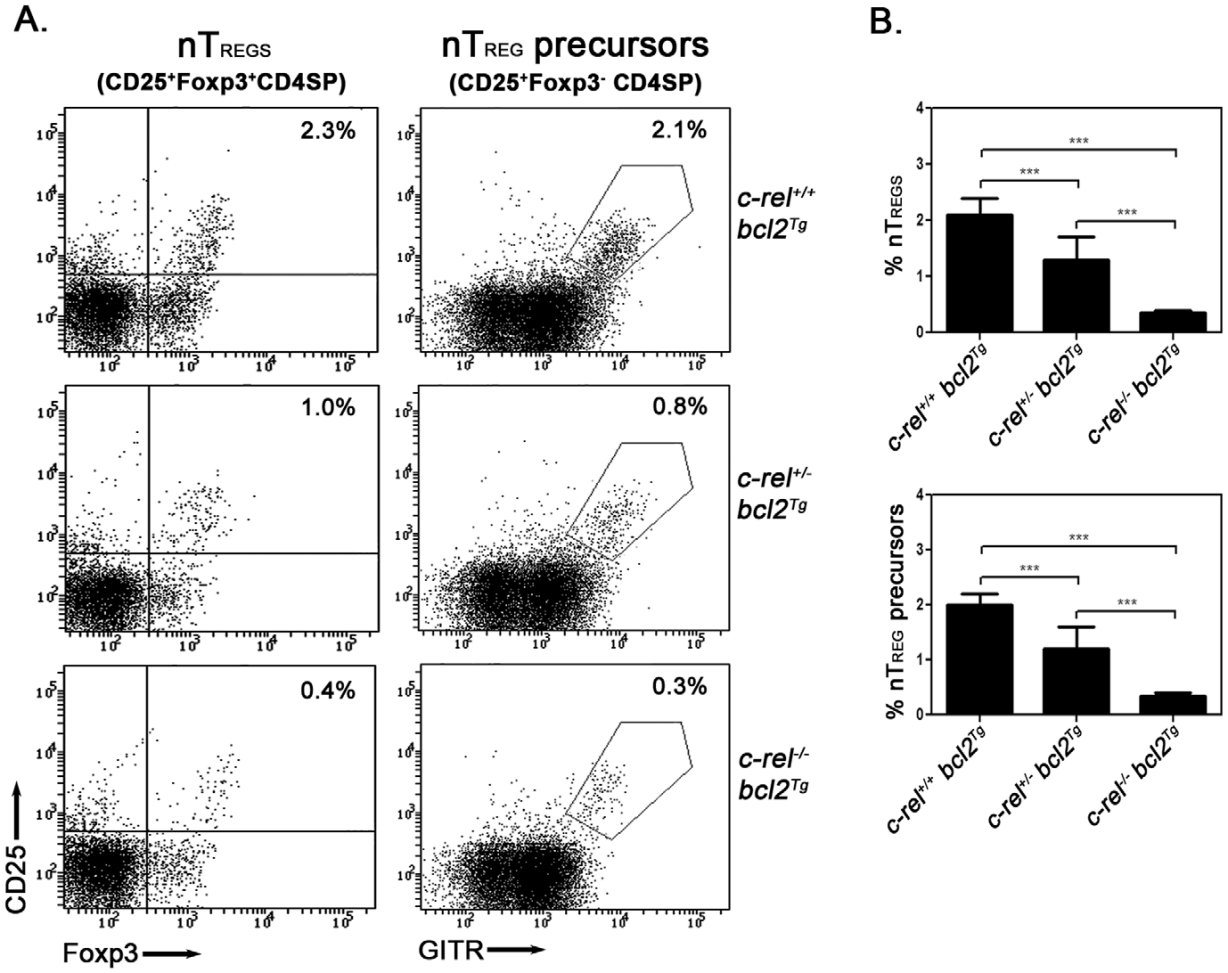
Bcl-2 transgene expression fails to rescue the deficit of nTreg precursors in c-Rel deficient mice. Thymocyte suspensions from *c-rel^+/+^bcl-2^Tg^*, *c-rel^+/−^bcl-2^Tg^* and *c-rel^−/−^bcl-2^Tg^* mice were fixed, permeabilized and stained with antibodies for CD4, CD8, CD25, GITR and Foxp3. Representative dot plots from one of three independent experiments are displayed with the percentage of cells in the relevant quadrants indicated. (A) Profiles of CD25 versus Foxp3 (gated on CD4 SP cells) and CD25 versus GITR (gated on Foxp3^−^CD4SP cells). Two mice of each genotype were analyzed in each experiment. (B) Percentages of thymic nTregs (CD25^+^Foxp3^+^CD4SP) and nTreg precursors (CD25^+^Foxp3^−^CD4SP) in *c-rel^+/+^bcl-2^Tg^*, *c-rel^+/−^bcl-2^Tg^* and *c-rel^−/−^bcl-2^Tg^* mice. The data represents the mean values (±SEM with ANOVA p value <0.0001) compiled from three independent experiments described in panel A.

Despite the failure of enforced Bcl-2 expression to rescue *c-rel^−/−^* thymic nTreg numbers [17], this study did not address the possibility that impaired regulation of the cell intrinsic survival pathway could contribute to the diminished nTreg precursor population in *c-rel^−/−^* mice. The c-Rel dependent induction of *A1* pro-survival gene expression in response to TCR signaling [30], coupled with the finding that TGFβ limits Bim-dependent apoptosis of thymic Treg precursors during negative selection [31], prompted us to reassess whether c-Rel influenced the survival of nTreg precursors. With enforced Bcl-2 expression shown to neutralize Bim-dependent apoptosis during thymocyte selection [32], we examined the impact a *bcl-2* transgene (*bcl-2^Tg^*) with a pan hemopoietic expression pattern [33] had on nTreg precursor numbers in *c-rel^+/−^* and *c-rel^−/−^* mice. Although enforced *bcl-2^Tg^* expression protected *wt, c-rel^+/−^* and *c-rel^−/−^* thymocytes from Bim-dependent growth factor withdrawal induced cell death in culture (results not shown), it did not correct the deficit of CD25^+^GITR^hi^Foxp3^−^CD4^+^ cells in *c-rel^+/−^* or *c-rel^−/−^* mice (Fig. 2A and 2B). This demonstrates that the reduced number of nTreg precursors that result from a loss of c-Rel function is not due to a defect in the cell intrinsic pro-survival pathway that operates during T cell selection.

### c-Rel regulates the IL-2 and IL-15 dependent induction of Foxp3 in nTreg precursors

**Figure 3:**
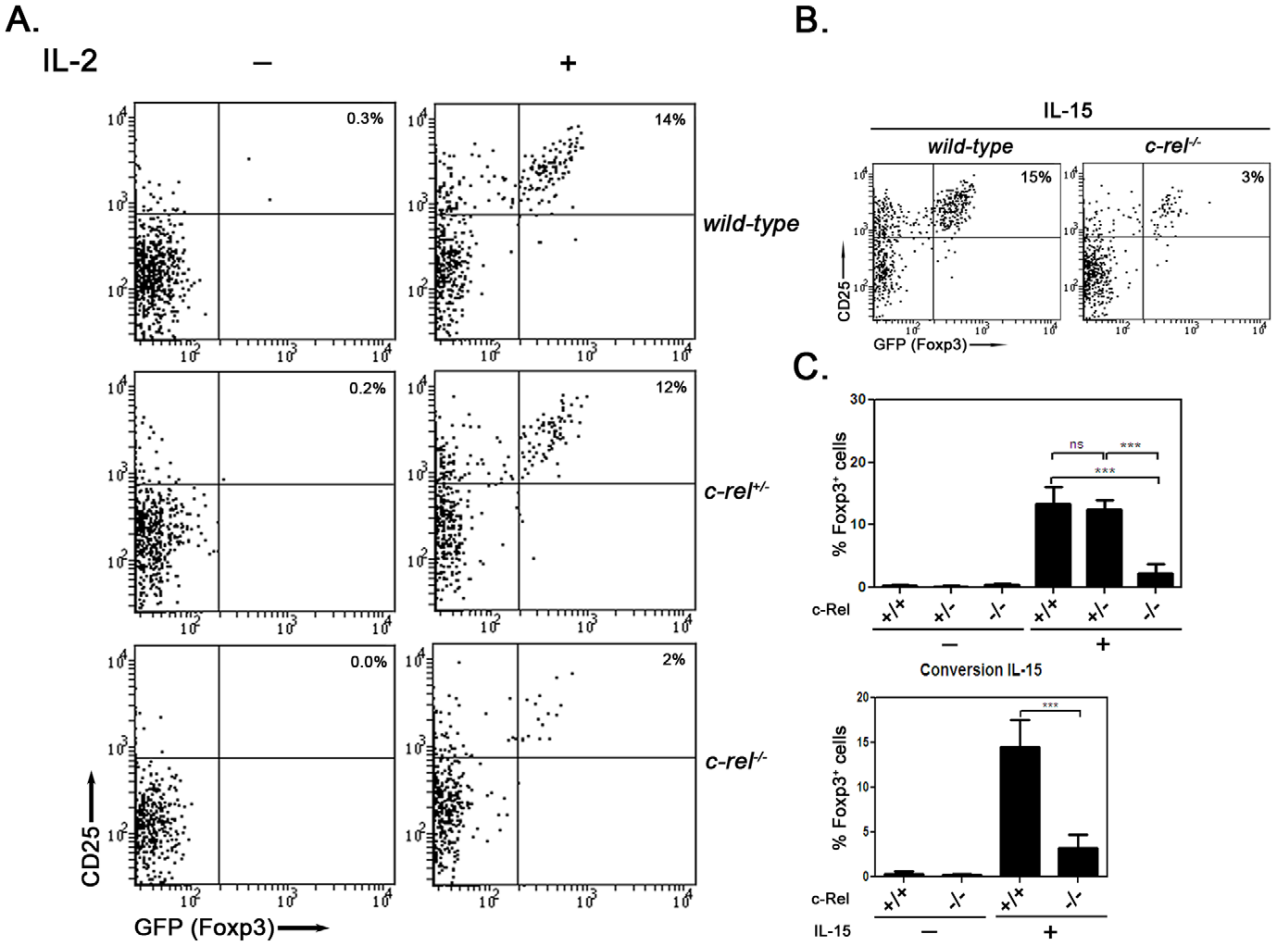
The IL-2 dependent differentiation of *c-rel^−/−^* nTreg precursors is impaired. Purified CD25^+^GITR^+^Foxp3^−^CD4^+^ nTreg precursors isolated from *foxp3^gfp^*, *c-rel^+/−^foxp3^gfp^* and *c-rel^−/−^foxp3^gfp^* mice cultured for 24 hrs in the absence or presence of IL-2 or IL-15 were analysed for Foxp3 expression (GFP^+^) using flow cytometry. In each case representative dot plots from one of six independent experiments are shown with the percentage of CD25^hi^Foxp3^+^ cells indicated. (A) IL-2 stimulation (B) IL-15 stimulation (C) Percentages of CD25^hi^Foxp3^+^ cells developing in cultures of cytokine treated *wt*, *c-rel^+/−^* and *c-rel^−/−^* thymic nTreg precursors (CD25^+^Foxp3^−^CD4SP cells). The data represents the mean values (±SEM with ANOVA p value <0.0001) compiled from six experiments described in panels A and B.

While c-Rel is necessary for the generation of nTreg precursors, emerging evidence that it may also be important in regulating Foxp3 expression during nTreg differentiation [18], [19], [20] prompted us to determine if c-Rel influences the cytokine dependent generation of Foxp3^+^ cells from Foxp3^−^ precursors. This was assessed by monitoring the IL-2 and IL-15 dependent differentiation of *c-rel^−/−^* nTreg precursors into Foxp3^+^ cells in culture. Initially, equivalent numbers of CD25^+^GITR^hi^Foxp3^−^CD4^+^ thymocytes isolated from *foxp3^gfp^* and *c-rel^−/−^foxp3^gfp^* mice were cultured for 24 hrs in 100 U/ml of IL-2 as previously described [11], [34]. The frequency of un-stimulated (media alone) nTreg precursors of both genotypes that express Foxp3 was <0.5% (Fig. 3A, 3C), confirming a need for IL-2 signaling in the induction of Foxp3. While IL-2 induced Foxp3 expression in ∼14% of cultured *wt* nTreg precursors, a frequency consistent with published reports [11], by contrast only 2 to 3% (±1.5 SEM) of *c-rel^−/−^* CD25^+^GITR^hi^Foxp3^−^CD4^+^ nTreg precursors up-regulated Foxp3 in response to IL-2 stimulation. Furthermore, although c-Rel controls the generation of nTreg precursor numbers in a gene copy dependent manner, *c-rel^+/−^* nTreg precursors unlike their *c-rel^−/−^* counterparts differentiate into Foxp3^+^ cells as efficiently as *wt* precursors following IL-2 stimulation (Fig. 3A and 3C). This difference in how *c-rel* copy number influences IL-2 dependent and independent nTreg differentiation indicates that c-Rel controls these two developmental steps via distinct mechanisms. The reduction in the frequency of *c-rel^−/−^* nTreg precursors able to up-regulate Foxp3 in response to IL-2 stimulation was not due to the increased death of these cells (Figure S1), nor was this defect IL-2 concentration dependent, given a 100-fold range in IL-2 levels (50 to 5,000 U/ml) still failed to increase Foxp3^+^ cell numbers (Figure S2). An inability to correct this cytokine dependent step by extending the period of IL-2 stimulation up to 48 hrs (results not shown) also established that the defect was not due to a delay in initiating Foxp3 expression. Finally, IL-15, another common γ chain (γc) cytokine that can promote the induction of Foxp3 expression [11], was unable to correct the cytokine dependent differentiation of *c-rel^−/−^* nTreg precursors (Fig. 3B and 3C). Collectively, these data show that *c-rel^−/−^* nTreg precursors have a cell intrinsic defect that impacts upon the IL-2 and IL-15 dependent induction of Foxp3.

### The expression of the high affinity trimeric IL-2 receptor complex on *c-rel^−/−^* nTreg precursors is normal

**Figure 4:**
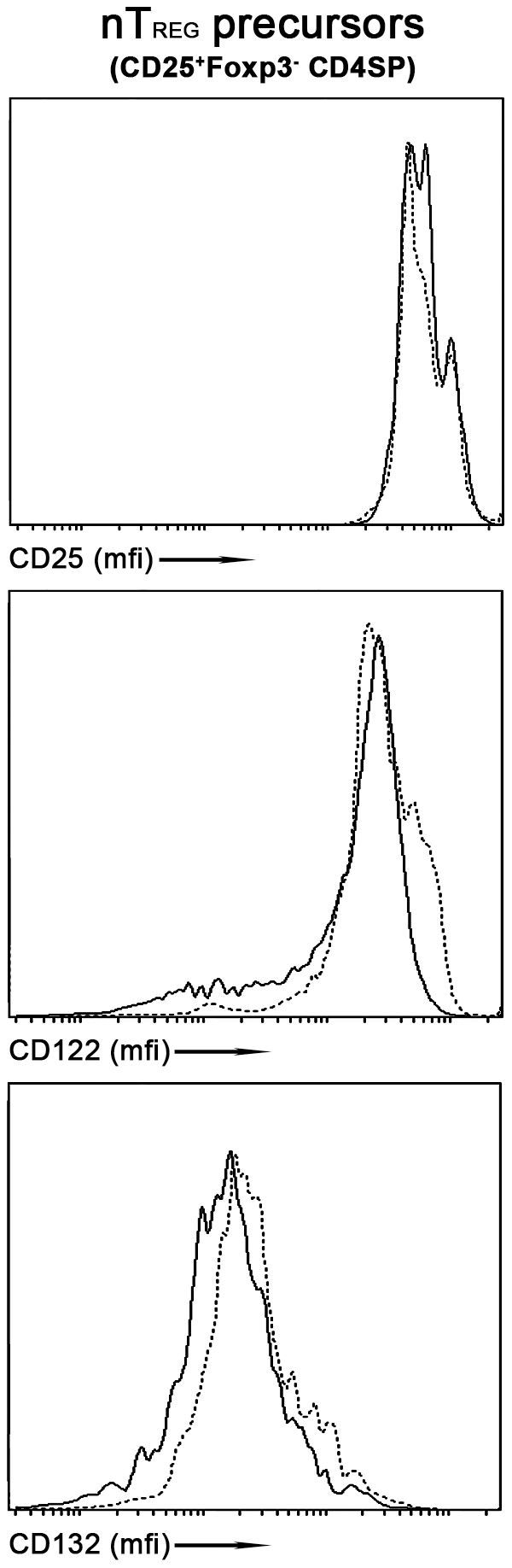
IL-2 receptor expression on nTreg precursors. Total thymocytes isolated from *foxp3^gfp^* and *c-rel^−/−^foxp3^gfp^* mice were pre-enriched for CD4 SP cells using a CD8 depletion strategy described in Methods, then stained with antibodies for CD4, GITR, CD25, CD122 and CD132. Histograms show expression levels (mean fluorescence intensity or mfi) of the CD25, CD122 and CD132 chains of the IL-2 receptor on *wt* (solid line) and *c-rel^−/−^* (broken line) nTreg precursors (GITR^hi^Foxp3^−^CD4SP cells). The data shown is representative of three independent experiments in which 3 mice of each genotype were used in each experiment.

Those cells within the CD25^+^GITR^hi^Foxp3^−^CD4^+^ thymocyte population that express high levels of the IL-2Rβ chain (CD122) account for the majority of nTreg precursors that possess a capacity to induce *foxp3* transcription in response to IL-2 stimulation [11]. With the NF-κB pathway implicated in controlling CD25 levels on conventional T cells [35], we compared the expression of CD25, CD122 and CD132 (IL-2R common γ-chain) on *wt* and *c-rel^−/−^* CD25^+^GITR^hi^Foxp3^−^CD4^+^ thymocytes (Fig. 4). CD25 and CD122 expression were equivalent on the *wt* and *c-rel^−/−^* nTreg precursor populations, while CD132 levels were slightly higher on *c-rel^−/−^* CD25^+^GITR^hi^Foxp3^−^CD4^+^ cells. Similar findings were made for the *wt* and *c-rel^−/−^* Foxp3^+^CD4^+^ thymocyte populations (results not shown). While fewer *c-rel^−/−^* CD25^+^GITR^hi^Foxp3^−^CD4^+^ thymocytes up-regulate CD25 in response to IL-2 stimulation (Fig. 3A), the reduction in CD25^hi^ cells was mainly restricted to the Foxp3^+^ population (Fig. 3A). A similar outcome was seen for *c-rel^−/−^* CD25^+^GITR^hi^Foxp3^−^CD4^+^ cells stimulated with IL-15 (Fig. 3B). Collectively our findings indicate that c-Rel does not appear to promote IL-2 dependent nTreg differentiation by controlling the expression of the IL-2 receptor complex. Instead, our data points to c-Rel regulating the IL-2 induction of Foxp3 in CD25^+^GITR^hi^Foxp3^−^CD4SP thymocytes via a mechanism that operates downstream of the IL-2 receptor.

### IL-2 dependent STAT5 phosphorylation is differentially regulated in nTreg precursors and nTregs by c-Rel

**Figure 5:**
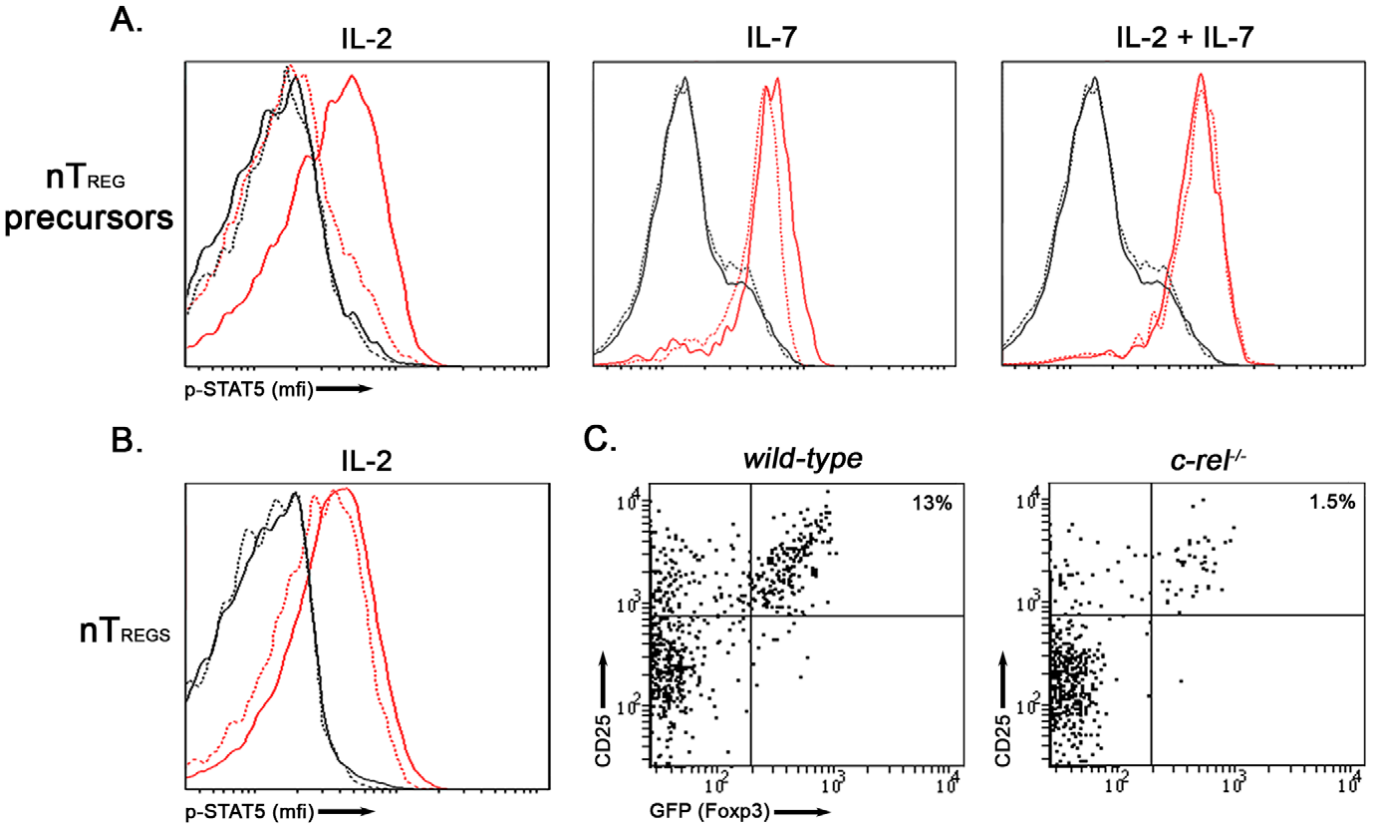
The IL-2 induced phosphorylation of STAT5 is impaired in *c-rel^−/−^* nTreg precursors. Purified nTreg precursors and nTregs isolated from *foxp3^gfp^* and *c-rel^−/−^foxp3^gfp^* mice stimulated with IL-2, IL-7 or IL-2 plus IL-7 for 30 minutes, were fixed, permeabilized and stained with antibodies to phospho-STAT5. (A) Phospho-STAT5 levels in cytokine stimulated *wt* and *c-rel^−/−^* nTreg precursors (CD25^+^Foxp3^−^CD4SP thymocytes). Histograms show expression levels (mfi) of phospho-STAT5 in *wt* (solid line) and *c-rel^−/−^* (broken line) nTreg precursors without (black lines) or in response to cytokine stimulation (red lines). (B) Phospho-STAT5 levels in IL-2 stimulated *wt* and *c-rel^−/−^* nTregs (CD25^+^Foxp3^+^CD4SP thymocytes). (C) Percentages of CD25^hi^Foxp3^+^ cells developing in cultures of *wt* and *c-rel^−/−^* nTreg precursors (CD25^+^Foxp3^−^CD4SP cells) co-stimulated with IL-2 plus IL-7. Experiments were performed as described in Fig. 3. The data shown in A and B are representative of three independent experiments, using 3 mice of either genotype in each experiment, while the data shown in panel C is based on three independent experiments.

A number of transcription factors including NFAT, Ets-1 and STAT5 are important for the induction and maintenance of *foxp3* expression [36], [37], [38]. In the case of STAT5, its IL-2 dependent phosphorylation by JAK is an essential requirement for activating this transcription factor [39]. Importantly in the context of this study, the NF-κB pathway has been shown to regulate STAT5 phosphorylation in conventional CD4 T cells [40] and CD4 Tregs [11] stimulated with common γ-chain cytokines. To determine if the reduced capacity of IL-2 to convert *c-rel^−/−^* nTreg precursors into Foxp3^+^ cells was associated with impaired STAT5 activation, IL-2 induced STAT5 phosphorylation was examined in *wt* and *c-rel^−/−^* CD25^+^GITR^hi^Foxp3^−^CD4^+^ thymocytes. Whereas IL-2 induced STAT5 phosphorylation in *wt* nTreg precursors, STAT5 was not phosphorylated in *c-rel^−/−^* CD25^+^GITR^hi^Foxp3^−^CD4^+^ cells (Fig. 5A). In contrast to nTreg precursors, levels of IL-2 induced STAT5 phosphorylation were equivalent in *wt* and *c-rel^−/−^* thymic Foxp3^+^ nTregs (Fig. 5B). These findings indicate that IL-2 induced STAT5 phosphorylation is differentially regulated in nTreg precursors and Foxp3^+^ nTregs by c-Rel. IL-7, another common chain cytokine that efficiently phosphorylated STAT5 in *wt* and *c-rel^−/−^* nTreg precursors (Fig. 5A) but alone cannot induce Foxp3 expression. But when combined with IL-2 induced normal levels of STAT5 phosphorylation in *c-rel^−/−^* nTreg precursors (Fig. 5A) still failed to correct the cytokine dependent defect in Foxp3 expression (Fig. 5C). These findings indicate that impaired IL-2 induced STAT5 phosphorylation is not the sole reason for the reduced IL-2 dependent conversion of *c-rel^−/−^* nTreg precursors into Foxp3^+^ cells.

### c-Rel is restricted to the cytoplasm of nTreg precursors and is not mobilized to the nucleus by IL-2 signaling

**Figure 6:**
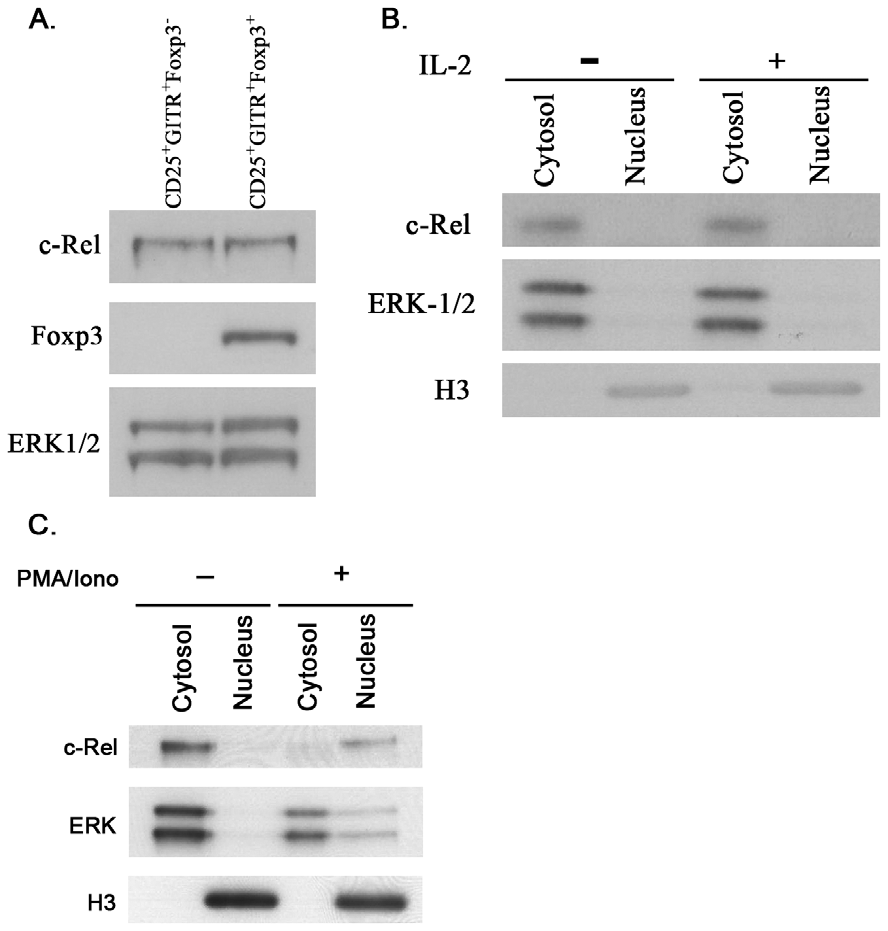
c-Rel expression in nTreg precursors. c-Rel expression in nTreg precursors and nTregs. (A) Whole cell lysates from equivalent numbers of purified nTreg precursors and nTregs were analysed by Western blotting for c-Rel, Foxp3 and ERK (loading control) expression. The data is representative of three independent experiments. Purified nTreg precursors cultured in the absence or presence of IL-2 (B) or PMA plus ionomycin (C) for 2 hrs were subjected to sub-cellular fractionation and Western blots performed on nuclear and cytosolic fractions for c-Rel, ERK (cytosolic loading control) and histone H3 (nuclear loading control) expression. The data shown in panels B and C is representative of three independent experiments.

The finding that c-Rel is required for the development of nTreg precursors and the IL-2 dependent differentiation of these cells into Foxp3^+^ nTregs indicated that c-Rel must be expressed in nTreg precursors. This was confirmed by performing Western blotting on whole cell extracts isolated from CD25^+^GITR^hi^Foxp3^−^CD4^+^ thymocytes, which like Foxp3^+^ nTregs [17] express high levels of c-Rel (Fig. 6A). However, an analysis of c-Rel levels in the cytoplasmic and nuclear fractions from nTreg precursors demonstrated that c-Rel was restricted to the cytosol of nTreg precursors (Fig. 6B). This indicates that c-Rel activity is not essential for the maintenance of these cells and instead is required at a developmental stage preceding the formation of nTreg precursors.

While a need for c-Rel function during the cytokine induction of Foxp3 in nTreg precursors infers that IL-2 signaling promotes c-Rel activation in these cells, Western blot analysis of nuclear fractions from IL-2 activated nTreg precursors clearly demonstrates that IL-2 does not induce nuclear c-Rel expression in these cells (Fig. 6B). The inability of IL-2 to mobilize c-Rel to the nucleus of nTreg precursors is not because c-Rel is refractory to activation in these cells, as phorbol ester plus ionomycin co-stimulation readily induced the nuclear expression of c-Rel in nTreg precursors (Fig. 6C). Instead these data indicate that the influence c-Rel exerts on the IL-2 dependent induction of Foxp3 expression is determined earlier in development at a stage that precedes the formation of nTreg precursors.

## Discussion

The differentiation of nTreg cells in the thymus proceeds via a two step process that first involves the TCR and CD28 dependent generation of CD25^+^GITR^hi^Foxp3^−^CD4^+^ nTreg precursors followed by the IL-2 or IL-15 induced maturation of these cells into Foxp3^+^ nTregs [11]. Here we establish that the c-Rel transcription factor is necessary for both the generation of nTreg precursors and the subsequent IL-2 induction of Foxp3. c-Rel appears to exert different levels of control over these two developmental steps, with the formation of nTreg precursors but not the cytokine induced expression of Foxp3 controlled by a c-Rel dose dependent mechanism. Despite a requirement for c-Rel in the IL-2 induction of Foxp3, this step proceeds independently of nuclear c-Rel expression and is consistent with a differentiation model in which c-Rel generates a state of transcriptional permissiveness during the development of nTreg precursors that promotes the ensuing IL-2 induced maturation of these cells into Foxp3^+^ nTregs.

Previous studies had shown that c-Rel is required for the generation of most Foxp3^+^ nTregs in the thymus [17], [18], [19], [20]. Here we show that the frequency of CD25^+^GITR^hi^Foxp3^−^CD4^+^ thymocytes, a population highly enriched for nTreg precursors is also reduced 5 to 6-fold in *c-rel^−/−^* mice. This finding confirms a recent report [21] indicating that c-Rel is required for the generation of nTreg precursors. Consistent with its regulation of nTreg precursor development, we demonstrate that c-Rel is expressed at high levels in CD25^+^GITR^hi^Foxp3^−^CD4^+^ cells. With c-Rel expressed at low levels in CD4^+^CD8^+^ thymocytes [17] but up-regulated in nTreg precursors, the induction of c-Rel during nTreg differentiation is most likely linked to T cell selection. This proposition is supported by the finding that c-Rel is induced in CD69^hi^TCRβ^hi^CD4^+^CD8^+^ thymocytes [41], cells that have recently undergone positive selection. Interestingly, the generation of nTreg precursors and consequently Foxp3^+^ nTregs is controlled in a *c-rel* copy dependent fashion. c-Rel and other NF-κB regulated cellular processes are not normally subjected to haplo-insufficient control [27], which indicates that c-Rel levels are limiting during the development of nTreg precursors.

It remains to be determined exactly which signals regulate c-Rel expression and its activation during the development of nTreg precursors. Although c-Rel is activated in conventional CD4 T cells by TCR [42] and CD28 [43] signaling, a report that TCR induced T cell proliferation, like nTreg precursor formation is controlled by c-Rel via a haplo-insufficient mechanism [44] indicates that TCR rather than CD28 signaling regulates the c-Rel dependent generation of nTreg precursors. The localization of c-Rel to the cytoplasm of nTreg precursors is indicative of transcriptional inactivity. While we cannot rule out that low amounts of c-Rel are present in the nucleus of nTreg precursors at levels sufficient to regulate transcription, this seems unlikely given the numerous signals that activate c-Rel in different cell types induce readily detectable levels in the nucleus. Instead we favor a model in which c-Rel is required for the differentiation of nTreg precursors rather than maintaining a function in these cells. Nevertheless, the continued expression of c-Rel in nTreg precursors points to these cells needing to retain a capacity to activate c-Rel. This could reflect some remaining developmental flexibility in CD25^+^GITR^hi^Foxp3^−^CD4SP thymocytes or the need to prepare nTregs for immune responsiveness once the process of thymic differentiation is complete.

Here we show that c-Rel is also required for the second major step in nTreg differentiation, the cytokine induction of Foxp3 expression in nTreg precursors. In the absence of c-Rel, only 2–3% of cultured CD25^+^GITR^hi^Foxp3^−^CD4^+^ thymocytes up-regulate Foxp3 in response to IL-2 or IL-15 stimulation. In those *c-rel^−/−^* nTreg precursors that induce Foxp3, its levels in these cells are normal, indicating that c-Rel controls the capacity of an nTreg precursor to induce Foxp3 transcription rather than determine absolute levels of Foxp3 expression. Unlike the generation of nTreg precursors which c-Rel regulates in a haplo-insufficient fashion, the c-Rel dependent control of IL-2 induced Foxp3 expression is not influenced by *c-rel* copy number. This indicates that the events c-Rel controls during these two distinct phases of nTreg differentiation exhibit a differential dependence on the cellular levels of this transcription factor. It also establishes that reduced numbers of nTregs in *c-rel^+/−^* mice reflect the role of c-Rel in generating nTreg precursors rather than a diminution of the frequency with which precursors convert into Foxp3^+^ nTregs.

Exactly how c-Rel controls the induction of Foxp3 expression in nTreg precursors remains unclear. Although multiple sequences within the *foxp3* locus bind c-Rel [18], [19], [20], of these, only CNS3 has been shown to control the frequency of Foxp3^+^ cells that develop in the thymus [20]. Like *c-rel^−/−^* mice [17], the *foxp3^ΔCNS3/ΔCNS3^* mutant displays a 5-fold reduction in thymic Foxp3^+^ nTregs [20], with those remaining nTregs expressing normal levels of Foxp3. While this data and the findings presented here provide compelling evidence that c-Rel regulates the induction of *foxp3* transcription through CNS3, it remains to be established whether c-Rel controls *foxp3* expression by directly binding to CNS3, or instead regulates another transcription factor that binds this element or other sites within the *foxp3* locus. The finding that c-Rel activates STAT5 by controlling its IL-2 induced phosphorylation, certainly lends support to the notion that c-Rel promotes the induction of *foxp3* expression by regulating other transcription factors. However, the inability of IL-7 to correct IL-2 induced Foxp3 expression in *c-rel^−/−^* nTreg precursors despite rescuing the STAT5 phosphorylation defect in these cells, indicates that c-Rel controls multiple events required for the induction of Foxp3.

Although the IL-2 induction of Foxp3 expression and STAT5 phosphorylation in nTreg precursors is c-Rel dependent, IL-2 does not promote the nuclear translocation of c-Rel in these cells. While it remains possible that c-Rel is constitutively expressed in the nucleus of nTreg precursors at levels below detection, this scenario seems unlikely. Instead the following models are offered as explanations for how c-Rel can regulate the IL-2 induced maturation of nTregs without needing to re-activate c-Rel in nTreg precursors. We propose that c-Rel remodels the chromatin structure of loci such as *foxp3* at a stage in thymocyte differentiation preceding the development of nTreg precursors, which then influences the subsequent activation of these genes by other IL-2 regulated transcription factors. Alternatively, c-Rel could generate a transcriptional memory in nTreg precursors through the partial assembly of an RNA polymerase II complex on the promoters of key target genes that are later activated by IL-2 signaling. This mechanism accounts for how RelA can promote the delayed induction of *inos* transcription in *L.monocytogenes* infected macrophages [45]. Future studies aimed at identifying those genes regulated by c-Rel in nTreg precursors and Foxp3^+^ nTregs will help to shed additional light on the mechanisms by which c-Rel controls the sequential differentiation of nTregs in response to TCR and cytokine signaling.

## Materials and Methods

### Mice

All experimental mice are on a C57BL/6 background and were 5 to 9 weeks of age. *foxp3^gfp^* [26], *c-rel^−/−^* [44], and *vav-bcl2* transgenic (*bcl-2^Tg^*) [33] mice were maintained as inbred strains. *c-rel^+/−^foxp3^gfp^* and *c-rel^−/−^foxp3^gfp^* mice were generated by intercrossing *c-rel^−/−^* and *foxp3^gfp^* mice, while *c-rel^+/−^bcl-2^Tg^* and *c-rel^−/−^bcl-2^Tg^* mice were generated by intercrossing *c-rel^−/−^* and *bcl-2^Tg^* mice. Animals were housed in a specific pathogen-free animal facility. Experiments using mice were approved by the Alfred Medical Research and Education Precinct Animal Ethics Committee in accordance with guidelines of the National Health and Medical Research Council, Australia (Approval number E/0847/2009/F).

### Reagents and antibodies

Recombinant murine IL-2 and IL-15 were from Peprotech (Princeton, NJ) and murine IL-7 from R&D Systems (Minneapolis, MN). Phorbol-12-myristate-13-acetate (PMA) and Ionomycin were from Sigma. The following antibodies were used: anti-mouse c-Rel [46], PE or PerCP-Cy5.5-conjugated anti-CD4 (RM4-5; BD Pharmigen), APC-Cy7-conjugated anti-CD25 (PC61), APC-conjugated anti-GITR (DTA-1), Pacific Blue (PB)-conjugated anti-CD8 (53-6.7), PE-conjugated anti–mouse Foxp3 (FJK-16S), biotin-conjugated CD122 and biotin conjugated CD132. Streptavidin-conjugated PE-Cy7 was purchased from eBioscience. Anti-rat IgG conjugated microbeads were obtained from Polysciences.

### Flow cytometry

An analysis of CD25, CD122 and CD132 expression on nTreg precursors was done using thymocytes from *foxp3^gfp^* and *c-rel^−/−^foxp3^gfp^* mice stained with anti–CD4-PerCP-Cy5.5, anti-CD8-Pacific Blue, anti-GITR-APC, anti–CD25-PE, anti-CD122-biotin and anti-CD-132-biotin labelled antibodies. Biotin labelled antibodies were detected using Streptavidin-PE-Cy7. Intracellular Foxp3 stains were performed on thymocyte suspensions prepared from adult *bcl2^T^*, *c-rel^+/−^bcl2^T^* and *c-rel^−/−^bcl2^T^* mice. Thymocytes were first stained with anti–CD4-PerCP-Cy5.5, anti-CD8-Pacific Blue and anti–CD25-APC-Cy7 antibodies, then intracellular Foxp3 stains were performed using PE-conjugated anti-Foxp3 antibodies (eBioscience) as previously described [17]. Stained cells were analysed using a FACSCalibur (Becton Dickinson) or LSR (Becton Dickinson) and the data analyzed using CellQuest Pro software (Becton Dickinson). CD25^+^GITR^+^Foxp3^−^CD4^+^ nTreg precursors and CD25^+^Foxp3^+^CD4^+^ nTregs were purified from *foxp3^gfp^* and *c-rel^−/−^foxp3^gfp^* mice by first employing an enrichment step that involved depleting CD8^+^ and CD4^+^CD8^+^ thymocytes. Anti-CD8 (Rat anti-mouse CD8α clone YTS-169) labelled thymocytes were incubated with anti-Rat IgG coupled microbeads. Following magnetic depletion, the unbound cell fraction was stained with anti–CD4-PerCP-Cy5.5, anti–CD8-Pacific blue, anti–CD25-APC-Cy7 plus anti-GITR-APC antibodies and CD25^+^GITR^hi^Foxp3^−^CD4^+^ and CD25^+^GITR^hi^Foxp3^+^CD4^+^ thymocytes isolated by flow cytometry using a FACSAria (Becton Dickinson). The purity of both sorted populations (CD25^+^GITR^hi^Foxp3^−^CD4^+^ precursors and CD25^+^GITR^hi^Foxp3^+^CD4^+^ nTregs) was typically >95%.

### Cell culture

Unless otherwise stated, *wt* and *c-rel^−/−^* CD25^+^GITR^hi^Foxp3^−^CD4^+^ nTreg precursors (5×10^3^ cells) isolated from *foxp3^gfp^* and *c-rel^−/−^foxp3^gfp^* mice by flow cytometry (>95% purity) were cultured in 96-well round bottom plates in 0.1 ml of Dulbecco's Modified Eagle Medium and 10% FBS, with or without cytokines (50 U/ml human IL-2, 50 ng/ml IL-7, IL-2 plus IL-7 or 100 ng/ml of IL-15) as previously described [11]. Foxp3 expression (GFP^+^ cells) was analyzed after 24 hours by flow cytometry using an LSR (Becton Dickinson).

### Intracellular phospho-STAT5 stains

Phospho-STAT5 was detected as described [47]. Following the depletion of CD8^+^ thymocytes from *foxp3^gfp^* and *c-rel^−/−^ foxp3^gfp^* mice, the remaining cells were incubated in serum-free DMEM and stimulated with human IL-2 (2500 U/ml) and/or murine IL-7 (50 ng/ml) for 30 mins. Cells were fixed at room temperature using 1.5% paraformaldehyde, washed and then resuspended in PBS/3% FCS. Cells were made permeable with methanol (20 min at 4°C), washed with PBS/3% FCS and incubated with Alexa Fluor-647-conjugated anti-phospho-STAT5 antibody for 30 min at room temperature. Cells were washed 3 times with PBS/3% FCS, incubated with 10% FCS for 10 min as a blocking step, stained with directly conjugated fluorescent antibodies for CD4, CD8, CD25 and GITR and analysed using an LSR (Becton Dickinson).

### Western blotting

Equal amounts of total cellular protein isolated from flow purified populations of CD25^+^GITR^hi^Foxp3^−^CD4^+^ and CD25^+^GITR^hi^Foxp3^+^CD4^+^ thymocytes were loaded on 10% Novex gels (Invitrogen) and then subjected to electrophoresis and Western blotting as previously described [48] using c-Rel-specific and anti-Foxp3 antibodies. Blots were stripped and reprobed with ERK antibodies. Western blots on nuclear and cytoplasmic extracts from CD25^+^GITR^hi^Foxp3^−^CD4^+^ and CD25^+^GITR^hi^Foxp3^+^CD4^+^ thymocytes prepared as previously described [46] were probed with antibodies to c-Rel, after which blots were stripped and reprobed with anti-ERK (Santa Cruz Biotechnology, Inc.) or histone H3–specific antibodies (Cell Signalling Technology).

### Statistical analysis

Data were subjected to one way ANOVA analysis followed by Tukey test using the GraphPad Prism software.

## Supporting Information

Figure S1 **The survival of** ***c-rel^−/−^*** **nTreg precursors in culture is normal.** The viability of nTreg precursors isolated from *foxp3^gfp^* and *c-rel^−/−^foxp3^gfp^* mice cultured in the presence of IL-2 for 24 hrs was measured as described [11]. Data is representative of 3 independent experiments.
(TIF)

Figure S2 **Different concentrations of IL-2 fail to rescue the Foxp3 expression defect in** ***c-rel^−/−^*** **nTreg precursors.** Purified CD25^+^GITR^+^Foxp3^−^CD4^+^ nTreg precursors isolated from *foxp3^gfp^* and *c-rel^−/−^foxp3^gfp^* mice cultured for 24 hrs in the absence or presence of 50, 500 or 5,000 U/ml of IL-2 were analysed for Foxp3 expression (GFP^+^) using flow cytometry. Shown is the percentage of CD25^hi^Foxp3^+^ cells (% of input cell numbers), with the data representing the mean values (±SEM) compiled from three independent experiments.
(TIF)

Table S1 **Absolute numbers of nTreg and nTreg precursors in the thymus.**
(DOCX)
