# Supplementary figures and images for: c-Rel Controls Multiple Discrete Steps in the Thymic Development of Foxp3+ CD4 Regulatory T Cells
Source: PLoS One. 2011 Oct 31;6(10):e26851. doi: 10.1371/journal.pone.0026851 (PMC3204987; doi:10.1371/journal.pone.0026851)

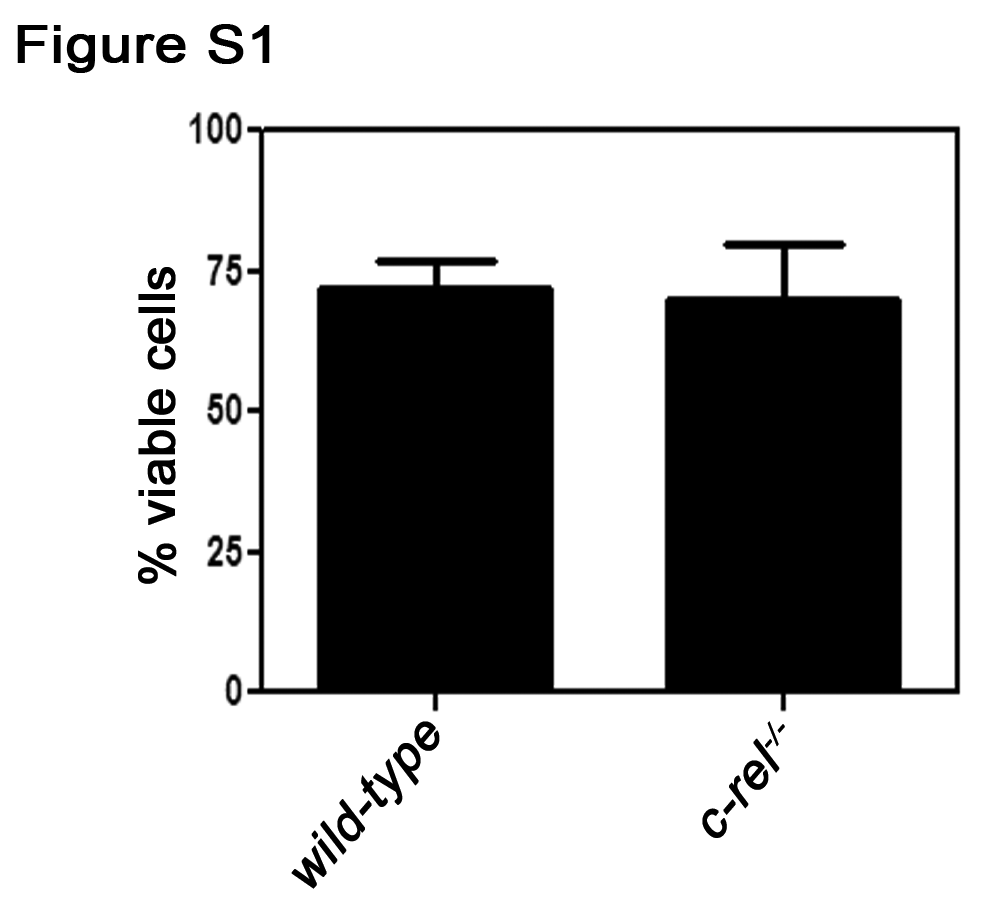

Supplement: Figure S1 — The survival of c-rel−/− nTreg precursors in culture is normal. The viability of nTreg precursors isolated from foxp3gfp and c-rel−/−foxp3gfp mice cultured in the presence of IL-2 for 24 hrs was measured as described [11]. Data is representative of 3 independent experiments. (TIF) [file pone.0026851.s001.tif]

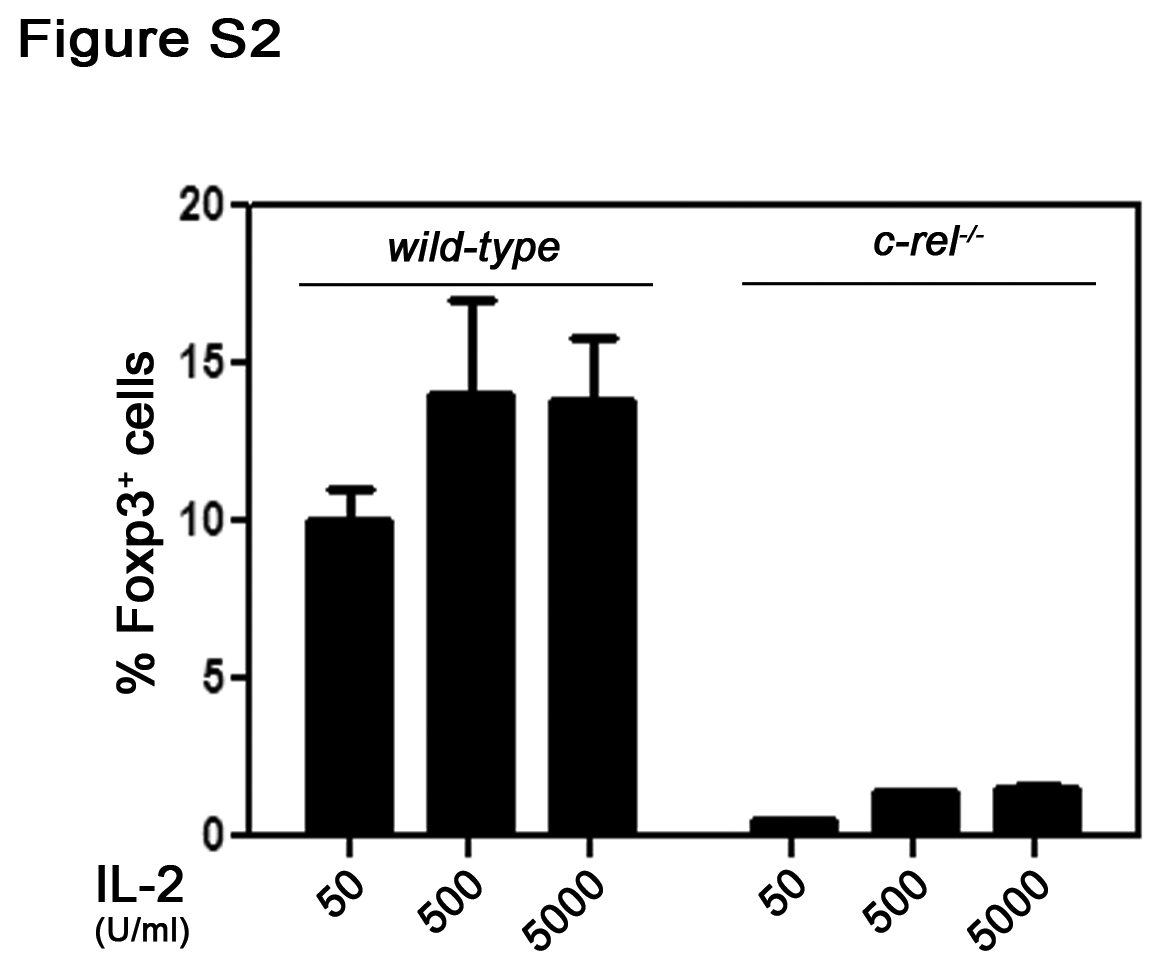

Supplement: Figure S2 — Different concentrations of IL-2 fail to rescue the Foxp3 expression defect in c-rel−/− nTreg precursors. Purified CD25+GITR+Foxp3−CD4+ nTreg precursors isolated from foxp3gfp and c-rel−/−foxp3gfp mice cultured for 24 hrs in the absence or presence of 50, 500 or 5,000 U/ml of IL-2 were analysed for Foxp3 expression (GFP+) using flow cytometry. Shown is the percentage of CD25hiFoxp3+ cells (% of input cell numbers), with the data representing the mean values (±SEM) compiled from three independent experiments. (TIF) [file pone.0026851.s002.tif]
