# Supplementary material for: c-Rel Controls Multiple Discrete Steps in the Thymic Development of Foxp3+ CD4 Regulatory T Cells
Source: PLoS One. 2011 Oct 31;6(10):e26851. doi: 10.1371/journal.pone.0026851 (PMC3204987; doi:10.1371/journal.pone.0026851)
Supplement: Table S1 — Absolute numbers of nTreg and nTreg precursors in the thymus. (DOCX) [file pone.0026851.s003.docx]

**Supplementary Table 1**: Absolute numbers of nTreg and nTreg precursors

in the thymus.

nTregs (×10^5^ ± SEM) nTreg Precursors (×10^5^ ± SEM)

*wild-type* 1.44 ± 0.35 1.06 ± 0.22

*c-rel^-/-^* 0.21 ± 0.04 0.15 ± 0.05

Data is cumulative of 10 *wild-type* and 6 *c-rel^-/-^* mice from three separate

experiments.
